# Chiari Malformation Type III: A Case Report and Review of Literature

**DOI:** 10.7759/cureus.14131

**Published:** 2021-03-26

**Authors:** Juan Fernando Ortiz, Samir Ruxmohan, Ammar Alli, Taras Halan, Ivan M Alzamora

**Affiliations:** 1 Neurology, Universidad San Francisco de Quito, Quito, ECU; 2 Neurology, Larkin Community Hospital, Miami, USA; 3 Medicine, Tishreen University Faculty of Medicine, Latakia, SYR; 4 Internal Medicine, Universitat de Barcelona, Barcelona, ESP; 5 General Medicine, Ternopil National Medical University, Ternopil, UKR

**Keywords:** encephalocele, chiari iii malformation

## Abstract

Chiari malformations (CMs) are a group of disorders involving deformities of the posterior fossa and hindbrain. There are seven types of CMs: 0, I, 1.5, II, III, IV, and V. CMIII is a very infrequent disorder characterized by low occipital or superior cervical encephalocele and inferior displacement of the brainstem. Here we present a unique case of CMIII associated with cleft palate, abnormal midbrain, and abnormal corpus callosum. CMIII is a very rare condition, which needs to be reported. This study primarily aims to compare our case to other cases of CMIII. We did not find another case with cleft palate and CMIII. There are only a few cases reported of CMIII. That is why it is vital to report each of these cases. Among reported CMIII cases, our case is unique regarding the co-occurrence of cleft palate. There seems to be an embryological link between these two conditions. However, cleft palate is a relatively common congenital defect, which means that the co-occurrence could be just a coincidence as well. Further research is warranted to broaden the information of this extremely rare syndrome.

## Introduction

The primitive brain has three main parts: the forebrain, the midbrain, and the hindbrain. The hindbrain later divides into the metencephalon and the myelencephalon. The metencephalon becomes the pons, and the myelencephalon becomes the cerebellum and medulla [1]. Chiari malformations (CMs) are anomalies of the hindbrain [2].

Deformities of the posterior fossa and hindbrain define CMs. Depending on the type of CM, three is herniation of the brainstem and cerebellum through the foramen magnum and the cervical spinal canal [3]. There are seven types of CMs [3]. Types I and II are the most common, whereas the other types are relatively rare [2].

CM type III (CMIII) is defined either by a low occipital encephalocele and superior cervical encephalocele. There is also inferior brainstem displacement, but it also can be absent. Displacement of cerebellar tissue posteriorly and inferiorly is also seen [4]. The natural course of CMIII is mainly unknown because of the rarity of the condition. The management and prognosis also vary from case to case. We present a unique case of CMIII with an associated cleft palate. We aim to compare the clinical features of our case with other cases of CMIII. Cleft palate seems to be a unique finding in our case. We also aim to discuss a possible link to this co-occurrence.

## Case presentation

Initial presentation

A 32-year-old Hispanic woman on her third pregnancy delivered a female child at 38 weeks by cesarean delivery. There were only three routine checkups during her pregnancy. The first control was at four months, and the mother found out she was pregnant approximately six weeks after conception. The mother consumed no prenatal vitamins, and folic acid was used after the first control.

Prenatal monitoring was abnormal for encephalocele, cleft palate, and single umbilical artery, which were observed in the second-trimester sonogram performed at four months of pregnancy. The mother was obese throughout the pregnancy, and group B streptococcus was unknown. During delivery, meconium-stained amniotic fluid was noted. The Apgar score was 7/9 and 7/9 at 1 minute and 5 minutes, respectively.

The neonate was then transferred to the neurology/neurosurgery neonatal intensive care unit for further management.

Physical examination

The head circumference was 32.5 cm above the encephalocele and 36 cm across the encephalocele. The eyes have small palpebral fissures bilaterally. The pupils were round and symmetric. Regular heart rhythm without heart murmurs was heard. A normal respiratory pattern was observed.

The extremities appeared well perfused. However, contractures in the upper and lower extremities were noted. The skin did not show a stigma of neurological disease. The neurological examination showed that the infant was easily responsive to tactile stimuli and had some extremities movement. The eyes opened spontaneously, and there was not an apparent visual or auditory problem.

Cranial nerves I-XII were intact. The arms and legs were held in a flexed position. There was an increased tone of the appendicular extremities, mainly in both legs. Regarding the sensory system, the infant responded easily to tactile stimuli.

Diagnostic workup

**Figure 1:**
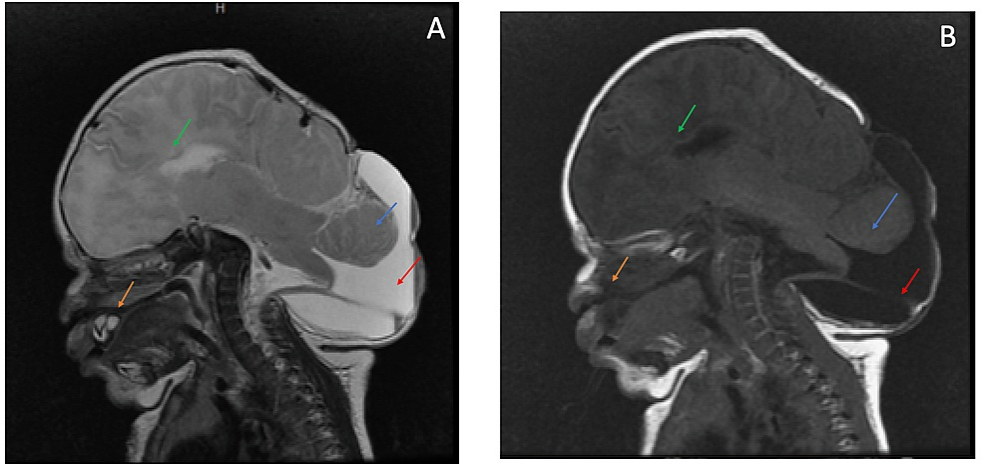
(A) MRI T2 (sagittal view). (B) MRI T1 (sagittal view). Both images show the low occipital/high cervical encephalocele. Green arrow: only the genu of the corpus callosum is seen, indicating partial agenesis. Blue arrow shows the brainstem in the encephalocele. Red arrow shows the hydrocele. Orange arrow shows the palate cleft defect. The midbrain is abnormal as well.

Magnetic resonance imaging (MRI) findings include low occipital and high cervical meningoencephalocele. There was hemosiderin deposition scattered throughout the herniated brain parenchyma, indicating prior hemorrhage. There was left parietal encephalocele, right periventricular areas of encephalomalacia, T1 hyperintensity in the median eminence, and cleft on the hard palate; only the genu of the corpus callosum was observed. The midbrain was abnormal. The third and fourth ventricles were not seen. Figure 1 shows an MRI of the patient.

**Figure 2:**
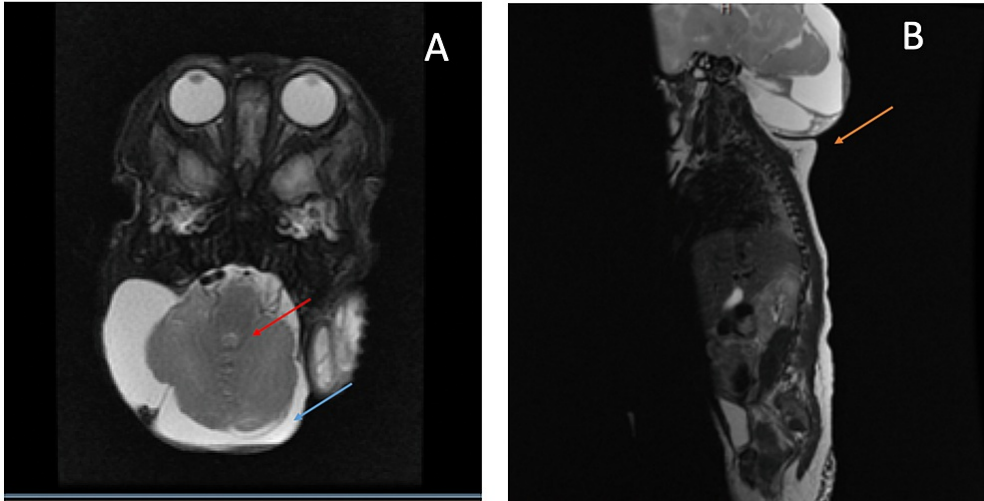
(A) MRI T2 (axial view). (B) MRI T2 (sagittal view). Red arrow shows the encephalocele (hypointense). Blue arrow shows the hydrocele (hyperintense). Orange arrow shows the low occipital/high cervical encephalocele from another angle.

Figure 2 shows additional findings from a different angle.

Follow-up

The neonate was breathing normally on room air, but she started having apneustic episodes on day 11 of birth. She was put on nasal intermittent positive pressure ventilation and then intubated. The neurology team was consulted and suggested that the apneustic episodes could be seizure-related. Hence, she was administrated prophylactic medication (levetiracetam). The encephalocele did not allow the collocation of the electroencephalogram leads.

The encephalocele was covered with a dressing that was changed daily. A ventricular reservoir was placed on the right side of the head for extraction of the cerebrospinal fluid (CSF).

The neurosurgery team was consulted, which decided not to operate because the brainstem was embedded in the encephalocele.

Impression and plan

The patient was diagnosed with CMIII. In addition, abnormalities of the corpus callosum and midbrain, and clef palate, which could be related or unrelated, were noted. Based on the findings, the neurosurgery team continued with the decision of not operating. Given her significant, irreversible, and devastating diagnosis and multiple complex congenital anomalies, the prognosis is poor. The neurological damage is progressive and permanent to a degree of absence of voluntary action and purposeful cognitive ability to interact in a meaningful way with her environment.

The neurology team will support the family’s decision to not resuscitate (DNR) status and withdraw life support if they choose to. Currently, the neonate is on mechanical ventilation, waiting for the family’s decision to withdraw life support and DNR status.

## Discussion

In this section, we will discuss the classification, pathogenesis, clinical features, and management of CMIII, and associate these findings with our case.

Classification

Table 1 details the different types of CMs [2,4,5].

**Table 1:** Classification of Chiari Malformation CSF, cerebrospinal fluid

Chiari Malformation	Clinical Features
Chiari 0	Abnormalities in the flow of CSF cause syringomyelia without causing herniation.
Chiari I	Cerebellar tonsils are 5 mm below the foramen magnum into the upper cervical canal. Chiari I is usually associated with syringomyelia. The prognosis is good.
Chiari 1.5	The same manifestations as Chiari I, with the addition of an elongated brainstem and fourth ventricle.
Chiari II	Herniation of the brainstem, cerebellum, and cerebellar tonsils. Chiari II is associated with myelomeningocele.
Chiari III	Herniation of the cerebellum with or without the brainstem. It also has low occipital and high cervical encephalocele.
Chiari IV	Cerebellar hypoplasia with associated myelomeningocele.
Chiari V	Cerebellar agenesis and herniation of the occipital lobe through the foramen magnum.

Our patient had the features of CMIII, with low occipital/upper cervical encephalocele, cleft palate, an abnormal midbrain, and partial agenesis of the corpus callosum.

Pathogenesis/embryology

The etiology of CMIII is mainly unknown. However, there is a theory that an incorrect neurulation during the ventricle extension causes an imperfect formation of the occipital area. Later, there is a prolapse of the cerebellum and the brainstem [5]. Another theory is that the ventricular system does not entirely descent due to abnormal neurulation. This abnormality caused a hypoplastic posterior fossa. Failure of the endochondral bone ossification is also a key factor for this condition [6]. According to Kiymaz et al., encephalocele is a mesoderm defect as well [7].

Our patient also had a cleft palate. Embryologically, the palate forms from mesoderm tissue and migration of neural crest cells. At week 4, the frontonasal prominence divides in the medial and lateral nasal processes. At week 6, the lateral nasal process fuses with the maxillary process bilaterally, forming the lower lip [8]. A fusion of the medial nasal processes forms the upper lip and palate. This process completes by week 10 [8].

Beyond sharing a mesoderm origin, it is difficult to establish an appropriate embryological link between CMIII and a cleft palate.

Clinical presentation

Only the genu of the patient’s corpus callosum was formed, the midbrain was abnormal, and the venous system was thought to be abnormal, but in the final reading, there were no abnormalities. According to Castillo et al., partial agenesis was reported in 6/9 cases of CMIII, aberrant venous drainage was observed in 4/9 cases of CMIII, and abnormal midbrain was seen in 8/9 cases [9]. The respiratory compromise was related to the degree of herniation and compromise of the brainstem [9].

According to Ivashchuk et al., only 57 CMIII cases have been reported [10]. CMIII is usually diagnosed in the prenatal period up to 14 years old [7]. The severity and prognosis of CMIII have been associated with the degree of brainstem embedded in the encephalocele. In these patients, 23 have high cervical/low occipital position encephalocele, 8 have cervical positions, 17 have low occipital positions, and 9 did not report the findings [10]. Our patient has a high cervical/low occipital position encephalocele. We did not find cases with co-occurrence of cleft palate and CMIII.

The patient’s brainstem was embedded in the encephalocele, discouraging the neurosurgery team from performing the surgery. The most common features of CMIII are highlighted in Table 2 [10].

**Table 2:** Clinical Features of Chiari Malformation Type III

Encephalocele	Meningoencephalocele
Occipital bone defect	Cervical bone defect
Small posterior cranial fossa	Caudal displacement of the hindbrain
Hydrocephalus	Aberrant venous drainage
Syringomyelia	Respiratory failure
Abnormalities of the midbrain	Herniation

Cleft palate is a relatively common birth defect observed in ½,000 cases, and the co-occurrence of CMIII and cleft palate could only be a coincidence [11]. Because the embryology of CMIII is not well established, it is difficult to find a link. Cleft palate has been associated with other conditions such as Pierre Robin sequence and cardiovelofacial syndrome, but not with CM [11].

Prognosis and treatment

CMIII is not necessarily fatal. Some patients may have productive lives [10]. However, patients usually have a poor prognosis and significant deficits if they survive [12]. The treatment is the closure of the anomaly. In general, patients with herniation of cerebral tissue < 5 cm have a better prognosis. The amount of cerebral tissue in the encephalocele and respiratory compromise are poor prognostic factors [12]. Our patient had a great amount of cerebral tissue herniated in the encephalocele, there were multiple neurological anomalies, and her breathing was compromised. These factors led the neurosurgery team to decide not to operate.

## Conclusions

CMIII is a very rare syndrome. It has a very low prevalence; therefore, it is essential to report it. The main feature of CMIII is the low occipital/upper cervical meningoencephalocele. However, our patient also presented with a cleft palate, abnormal midbrain, and partial agenesis of the corpus callosum.

Most of the features of our patient have been reported except cleft palate. However, cleft palate is a relatively common congenital defect, which means that the co-occurrence of CMIII and cleft palate could be just a coincidence. The embryology of CMIII is not well established; therefore, it is difficult to find an embryological link with cleft palate.

CMIII cases need to be reported. This is a rare condition, which limits physicians because of the lack of information on CMIII.
